# Pannexin1: insight into inflammatory conditions and its potential involvement in multiple organ dysfunction syndrome

**DOI:** 10.3389/fimmu.2023.1217366

**Published:** 2023-08-30

**Authors:** Xiangyu Chen, Siyi Yuan, Liangyu Mi, Yun Long, Huaiwu He

**Affiliations:** Department of Critical Care Medicine, State Key Laboratory of Complex Severe and Rare Diseases, Peking Union Medical College Hospital, Chinese Academy of Medical Sciences, Beijing, China

**Keywords:** pannexin 1 (Panx1), sepsis, MODS, inflammasome, microcirculation

## Abstract

Sepsis represents a global health concern, and patients with severe sepsis are at risk of experiencing MODS (multiple organ dysfunction syndrome), which is associated with elevated mortality rates and a poorer prognosis. The development of sepsis involves hyperactive inflammation, immune disorder, and disrupted microcirculation. It is crucial to identify targets within these processes to develop therapeutic interventions. One such potential target is Panx1 (pannexin-1), a widely expressed transmembrane protein that facilitates the passage of molecules smaller than 1 KDa, such as ATP. Accumulating evidence has implicated the involvement of Panx1 in sepsis-associated MODS. It attracts immune cells via the purinergic signaling pathway, mediates immune responses via the Panx1-IL-33 axis, promotes immune cell apoptosis, regulates blood flow by modulating VSMCs’ and vascular endothelial cells’ tension, and disrupts microcirculation by elevating endothelial permeability and promoting microthrombosis. At the level of organs, Panx1 contributes to inflammatory injury in multiple organs. Panx1 primarily exacerbates injury and hinders recovery, making it a potential target for sepsis-induced MODS. While no drugs have been developed explicitly against Panx1, some compounds that inhibit Panx1 hemichannels have been used extensively in experiments. However, given that Panx1’s role may vary during different phases of sepsis, more investigations are required before interventions against Panx1 can be applied in clinical. Overall, Panx1 may be a promising target for sepsis-induced MODS. Nevertheless, further research is needed to understand its complex role in different stages of sepsis fully and to develop suitable pharmaceutical interventions for clinical use.

## 1 Introduction

Sepsis, a life-threatening condition triggered by a dysregulated immune response, arises from severe infections, trauma, burns, shock, and major surgeries. This global health concern accounts for approximately 50 million cases and 11 million deaths yearly (1). Among patients with severe sepsis, the development of MODS leads to a poor prognosis (2). Those diagnosed with sepsis and MODS exhibit significantly higher mortality and rehospitalization rates than those without MODS (3). To improve clinical management and devise novel therapeutics, it’s crucial to comprehend the mechanisms underlying MODS in sepsis. However, despite ongoing research, viable therapeutic targets for septic MODS have yet to be identified.

The Panx (pannexin) protein family is found in vertebrates, first identified in 2000 (4). Panx and hemichannel Cx (connexin) function as unselective channels in vertebrates. Initially considered a transmembrane channel due to extracellular glycosylation modification (5), Panx1 was later found to form intercellular cell-to-cell channels, indicating its role as a hemichannel (6). The Panx family comprises three members: Panx1, widely expressed in various tissues such as eyes, kidneys, liver, CNS (central nervous system), vascular endothelium, alveolar epithelium, and immune cells; Panx2, predominantly found in CNS with subcellular localization on intracellular membranes; and Panx3, exclusively detected in skin and osteoblasts (7, 8). Panx1 acts as a non-selective hemichannel, allowing the passage of molecules smaller than 1 KDa, including ATP (adenosine-triphosphate) and dye molecules (9). Its involvement spans various physiological and pathological processes across different cell types. Hypoxia and mechanical stress can activate Panx1 hemichannels on erythrocytes, leading to ATP release, which, in turn, dilates vascular endothelium through the purinergic signaling pathway (10, 11). In neurons under hypoxic and glucose-deficient conditions, NMDAR (N-methyl-d-aspartate receptor) signaling or K^+^ current activates SFK (Src kinase), which phosphorylates Panx1, resulting in agonistic toxicity and impaired neuronal recovery in inflammatory conditions (12, 13). Additionally, Panx1 involves tumor migration and metastasis, as mechanical forces on tumor cells induce the opening of Panx1 hemichannels on vascular endothelium, leading to ATP release that promotes metastasis (14). Elevated Panx1 levels are associated with a poor prognosis in certain cancers (15).

Studies have demonstrated Panx1’s role in inflammation (16, 17). At the organ level, Panx1 has been implicated in the inflammatory response in various organs, including the heart, brain, lungs, kidneys, and liver (18–22). Furthermore, Panx1’s involvement in developing systemic inflammatory disorders, such as sepsis, has been investigated (23, 24). However, it’s not clear whether Panx1 is potential as a therapeutic target for sepsis-induced MODS. This article aims to provide an overview of Panx1 in inflammation, immunosuppression, and microcirculation, as well as its role in the inflammatory damage of multiple organs. This article aims to shed light on Panx1’s potential therapeutic value against sepsis-induced MODS.

## 2 Panx1’s contribution to hyperinflammation

**Figure 1:**
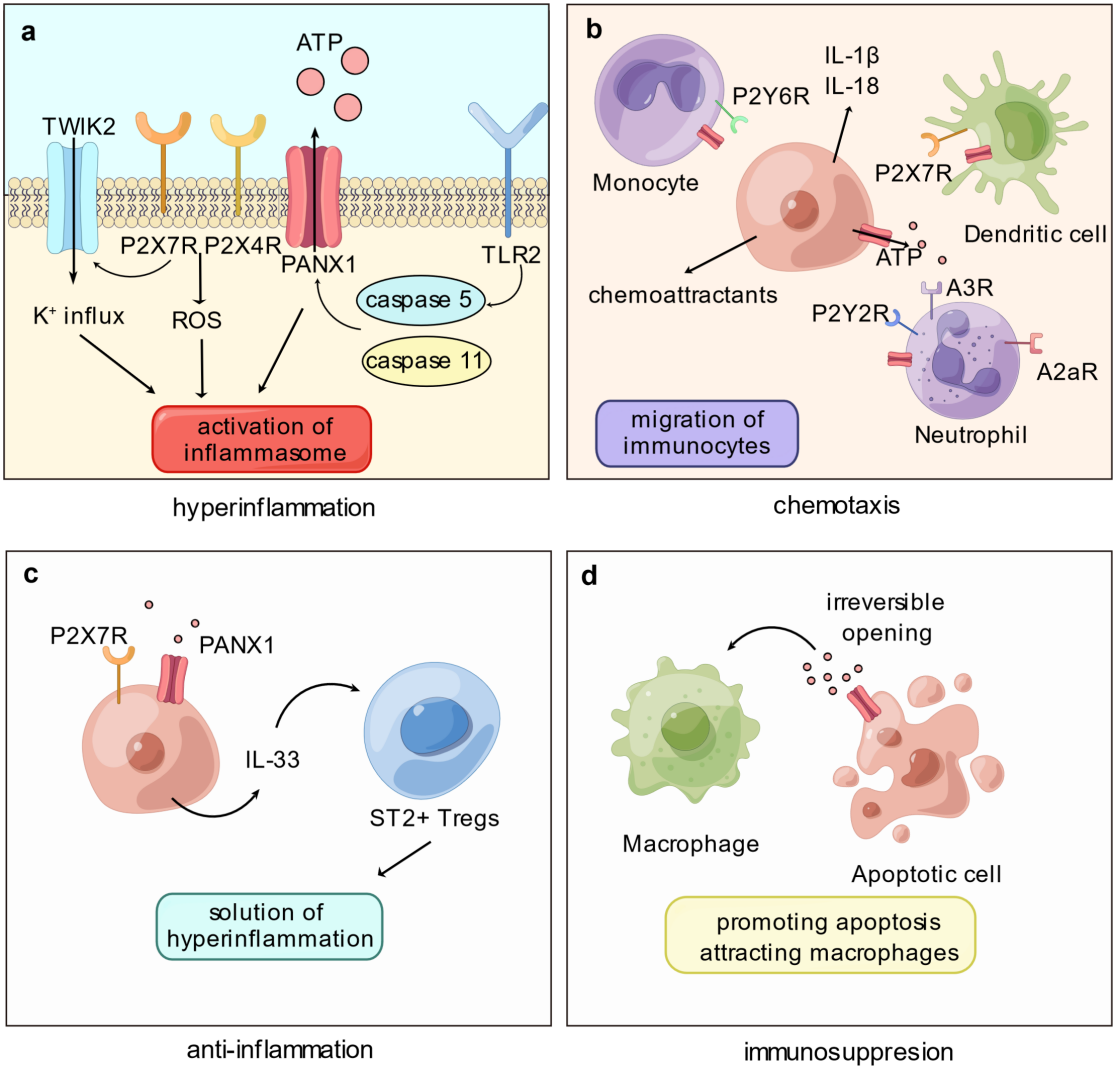
Panx1 participates in immune disorder by proinflammation and immunosuppression. **(A)** The proinflammatory role of Panx1 is associated with activation of the inflammasome. Cleavage of Panx1 by caspase proteins, such as caspase-5 or -11, opens Panx1 and leads to downstream signaling pathways that activate inflammasome: Purinergic stimulation on P2X_7_R and P2X_4_R promotes the production of cytoplasmic ROS; P2X_7_R mediates the K^+^ influx via TWIK2 channel. **(B)** Opening of Panx1 recruits immune cells mainly via the purinergic signaling pathway. ATP released via Panx1 attracts monocytes, neutrophils, and dendritic cells, meanwhile Panx1 on immune cells mediates migration. Release of proinflammatory molecules or chemoattractants promoted by Panx1 hemichannel activation, such as IL-1β and IL-18, certainly elevates immunocyte migration. **(C)** Panx1 contributes to immunosuppression by mediating the production of IL-33. As an immunoregulatory molecule, IL-33 binds ST2^+^ Tregs and assists resolution of hyperinflammation. **(D)** Activation of Panx1 hemichannel elevates apoptosis level of immune cells, promoting immune paralysis. Further, ATP released by Panx1 acts as “find-me” signals and attracts macrophages.

According to Sepsis-3 criteria, sepsis is characterized as a dysregulated host response to infection, wherein excessive inflammation and immunosuppression coincide; in cases where the body fails to effectively eliminate a high pathogen load, an exaggerated inflammatory response ensues, coupled with paradoxical anti-inflammatory regulation. The early phase of sepsis exhibits hyperinflammation involving multiple cell types and complex networks. While Panx1 is not directly engaged in the initial process, it plays a significant role by releasing signaling molecules like ATP, which can contribute to the proinflammatory response. Specifically, Panx1 promotes inflammasome activation (16) and facilitates the migration of immune cells (25) (**Figure 1**). It is also involved in releasing neutrophil extracellular traps via the purinergic signaling pathway (26, 27).

### 2.1 Panx1’s role in inflammasome activation

In sepsis, the delicate balance between protective and detrimental inflammation becomes crucial, as excessive inflammation can damage tissue. Inflammasome activation plays a vital role in hyperinflammation during sepsis, as it triggers the production and release of proinflammatory cytokines, such as IL-1β (Interleukin 1β) and IL-18 (Interleukin 18), and induces inflammatory cellular death, including pyroptosis and necroptosis. The inflammasome is a protein complex that responds to infection or damage. Cytoplasmic receptors like NLRP3 (NOD-like receptor thermal protein domain associated protein 3) recruit ASC (apoptosis-associated speck-like protein containing a caspase recruitment domain) to activate caspase-1 (28). In contrast, LPS (lipopolysaccharides) can activate caspase-11 in murine or caspase-4/5 in humans in a non-canonical manner, leading to the activation of NLRP3 and caspase-1 (29). Mature caspase-1 cleavages IL-1β and IL-18 into maturity, both of which play critical roles in the early phase of sepsis, with elevated levels exacerbating the inflammatory response (30). Moreover, downstream inflammatory cellular death can lead to tissue injury and further amplify inflammation in sepsis (31).

Panx1 has been found to promote inflammasome activation via the purinergic signaling pathway (16). However, the mechanism linking Panx1 to downstream NLRP3 activation has yet to establish fully. The prevailing hypothesis suggests that Panx1 activates inflammasome by releasing ATP and activating P2X_7_ receptors, which are ATP-gated ion channels. Although initially believed to be a K^+^ efflux channel (32), recent studies have shown that P2X_7_ receptors only allow the influx of Ca^2+^ and Na^+^ upon activation, and the K^+^ current via TWIK2 (two-pore domain weakly inwardly rectifying K^+^ channel 2), promoted by P2X_7_ receptors, activates NLRP3 (33). Moreover, inflammasome activation is associated with producing cytoplasmic ROS (reactive oxygen species), which is enhanced by P2X_7_ and P2X_4_ receptors upon ATP signaling from Panx1 (34, 35). Since P2X_7_ and P2X_4_ receptors form heterodimeric trimers, they are structurally and functionally interdependent (36, 37), suggesting that the inflammasome activation by Panx1 and P2X_7_ receptors may also involve P2X_4_ receptors.

In addition to NLRP3, Panx1 may also be involved in the activation of the non-canonical inflammasome, such as NLRP1, which is associated with the upregulation of M1 macrophages (the proinflammatory phenotype of macrophages) (38), as well as neuronal aging (39). Additionally, Panx1 has been found to promote AIM2 (absent in melanoma 2) activation, contributing to neuronal damage in conditions like subarachnoid hemorrhage (40), myocardial death in heart failure (41), pyroptosis of retinal cells due to ocular hypertension (42), and inflammatory response of Kupffer cells following hepatic ischemia-reperfusion injury (43). Furthermore, Panx1’s involvement in the non-classical pathway is evident, where murine caspase-11 cleaves the C-terminus of Panx1, leading to NLRP3 activation (21). Moreover, TLR2 interactions with human-derived caspase-5 can also lead to Panx1 cleavage and promote an inflammatory response (44, 45).

The role of Panx1 in inflammasome activation and pyroptosis has been a topic of debate among researchers. Some studies suggest that Panx1 likely plays a role in inflammasome activation, supported by experiments using Panx1 hemichannel inhibitors like probenecid, which have demonstrated inhibitory effects on inflammation responses (40, 46). However, this method may be limited, as probenecid inhibits P2X_7_ receptors (47). On the other hand, some experiments have demonstrated that inflammasome can still be activated in macrophages even in the absence of Panx1, suggesting that under certain conditions, Panx1 may not be a mandatory component for inflammasome activation (48, 49). To gain a comprehensive understanding, more extensive and detailed studies are required to fully elucidate the involvement of Panx1 in inflammasome activation and pyroptosis.

### 2.2 Panx1’s function in mediating immune cell chemotaxis

Chemokines, such as C5a and leukotrienes, play a pivotal role in orchestrating immune response by driving immune cells, especially neutrophils, from bone marrow or blood to sites of inflammation or organs during uncontrolled inflammatory responses (50). The degree of immune cell infiltration in organs strongly correlates with organ damage and mortality in sepsis. Consequently, interventions targeting chemotaxis hold promise as potential therapeutic strategies to mitigate organ injury and enhance survival rates (51–53). Immune cells, like neutrophils, employ an amoeboid mode of migration characterized by minimal adherence to the extracellular matrix, largely dependent on cytoskeletal contractility and mechanosensitive channels (54). Panx1 emerges as a crucial player in purinergic and Ca2+ signaling and cytoskeleton control within this complex regulatory network. This section provides a concise overview of Panx1’s involvement in chemotaxis, with more comprehensive insights available in Harcha’s review (17).

In the context of neutrophil migration, Panx1 localizes at the leading edge of the cellular membrane alongside F-actin (55). By releasing ATP, Panx1 indirectly influences neutrophil movement bidirectionally. The purinergic pathway, mediated by Panx1, exerts a dual role in regulating neutrophil behavior: ATP activates polarly distributed P2Y_2_ receptors, while adenosine, derived from ATP, stimulates A3 receptors, creating an autocrine regulatory network that enhances gradient sensitivity and sustains cell polarity (55, 56). At the rear of neutrophils, adenosine interacts with A2a receptors, counteracting agonistic signaling and triggering the cAMP/PKA pathway (57).

In DCs (dendritic cells), Panx1 collaborates with P2X_7_ receptors to form an autocrine loop. In the presence of elevated extracellular ATP levels, P2X_7_ receptors activate and induce Ca^2+^ influx, leading to Panx1 hemichannel opening and consequent ATP release. This process triggers the activation of CaMKII (calmodulin-dependent protein kinase II), ultimately altering cytoskeletal structure, and enabling rapid DC migration (25). Panx1 connects the purinergic pathway with Ca^2+^ signaling in DCs via CaMKII, which can directly open Panx1 hemichannels (58).

While monocytes and macrophages share a common origin and exhibit similar markers, the exact involvement of Panx1 in monocyte migration remains somewhat enigmatic. Monocytes in the bloodstream can differentiate into either DCs or macrophages. In an ATP-mediated autocrine loop, they release ATP to activate P2Y_6_ receptors in response to CCL2-induced (C-C motif ligand 2) chemotaxis (59). The specific role of Panx1 in monocyte migration has yet to be entirely elucidated, even though both Cx and Panx1 are expressed in these cells (60). Panx1 on apoptotic cells serves as a “find-me” signal by releasing ATP to attract macrophages. Nevertheless, it remains unclear whether Panx1 on macrophages also actively mediates chemotaxis. Prior investigations have suggested inhibiting Panx1 hemichannels can impede microglial migration (macrophages within the nervous system) and reduce their numbers at the injury site (61). However, this effect may be associated with reduced levels of pro-inflammatory cytokines (62). Further investigations are warranted to fully comprehend the intricate role of Panx1 in mediating immune cell chemotaxis.

## 3 Panx1’s involvement in immunosuppression

Sepsis presents a complex immune landscape characterized by both hyperactive inflammation and immunosuppression. During the late phase of sepsis, the body’s ability to eliminate pathogens is hindered by abnormal immune system suppression, resulting in inflammation-related immunosuppression. This factor significantly contributes to secondary infections and the development of MODS, which remains a primary cause of poor prognosis in septic patients (63). While Panx1 has been discussed for its role in fueling inflammatory responses, it also exhibits a dual function in the immune response, as it is involved in mediating Tregs (regulatory T cells) and promoting immune cell death. This suggests that Panx1 actively suppresses immune response (**Figure 1**).

### 3.1 Panx1’s anti-inflammatory effects through Treg regulation

In sepsis, an immunosuppressive state ensues, characterized by the release of anti-inflammatory cytokines, immune cell death, T-cell exhaustion, and elevated levels of immunomodulatory cells, notably Tregs (64). Recent research by Medina et al. has shed light on Panx1’s role in mediating Tregs and its capacity to curtail inflammation through the purinergic signaling pathway (65). While extracellular ATP acts as a danger signal to initiate the inflammatory response by activating the inflammasome and recruiting immune cells, its degradation into adenosine by ectonucleotidases CD39 and CD73 can help limit inflammation and regulate immune responses. Adenosines facilitate intercellular communication between Tregs and Teffs (effector T cells), inhibiting Teff cell proliferation within the airway. This study underscores the significance of Panx1-dependent crosstalk between Treg and Teff cells in mitigating inflammation.

The Panx1-IL-33 (interleukin-33) axis is crucial in resolving excessive inflammation in the liver (66). Upon LPS stimulation, hepatic cells release ATP, activating P2X_7_ receptors and producing IL-33, a member of the IL-1 protein family that is not activated by caspase-1-dependent cleavage. This cytokine fosters the recruitment of liver-infiltrating Tregs expressing its receptor, ST2, thereby resolving hyperinflammation in sepsis (66). Notably, in liver transplantation models infected with MRSA (methicillin-resistant *Staphylococcus aureus*), the Panx1-IL-33 axis is distinct in enhancing bacterial elimination. Panx1-mediated purinergic signals activate P2X_2_ receptors, leading to IL-33 production in hepatocytes. Consequently, this axis recruits macrophages and neutrophils, effectively reducing MRSA infection (67). These studies highlight the diverse mechanisms by which the Panx1-IL-33 axis regulates immune responses in the liver, with low levels of Panx1 potentially limiting its immunoregulatory effects.

Moreover, studies have suggested that Panx1 may also influence the population of infiltrating phagocytes during peritonitis development, further emphasizing its involvement in immunomodulation (68). As previously mentioned, Panx1 acts as an upstream modulator, influencing pro-inflammatory and anti-inflammatory responses via the purinergic pathway. However, given the intricate nature of this regulatory network, the mechanisms underlying Panx1’s anti-inflammatory effects are not yet fully elucidated, underscoring the need for further research in this area.

### 3.2 Panx1’s contribution to immunosuppression through immunocyte death

In sepsis, the impairment of effector immune cells through various forms of programmed cell death exacerbates the immunosuppressive state. Pyroptosis, apoptosis, autophagy, and ferroptosis have been observed in immune cells during sepsis, collectively contributing to compromised immune function and hindering the body’s ability to combat infections (64). While the relationship between Panx1 and autophagy has yet to be definitively established (69), a study by Su et al. has reported a potential role of Panx1 in promoting ferroptosis of human renal cells during ischemia-reperfusion injury of the kidney (70). However, further investigations are needed to elucidate these connections fully.

Pyroptosis, a lytic and pro-inflammatory type of cell death, is characterized by cell swelling, pore formation, and rapid disruption of membrane integrity. It is activated by the inflammasome (71), and as discussed earlier, Panx1 may promote pyroptosis by activating the inflammasome (35, 72). Yang et al. reported that Panx1 promotes pyroptosis of macrophages upon LPS challenge, where cytosolic LPS induces caspase-11-dependent cleavage of Panx1, activating downstream P2X_7_ receptors (31). High levels of pyroptosis in immune cells impair the host’s ability to eliminate infections and worsen the overall condition (73). Notably, ablation of PANX1 has been shown to reduce the level of pyroptosis and enhance resistance to sepsis.

In contrast, apoptosis is a non-inflammatory type of programmed cellular death frequently observed during sepsis. During apoptosis, the C-terminus of Panx1 undergoes irreversible cleavage, a critical functional structure required for Panx1 activity (74). The effector proteins caspase-3/7 shear the C-terminus of Panx1, particularly the DVVD region at the C-terminus, as reported by Chekeni et al. (75), resulting in a sustained increase in Panx1 hemichannel activity, ultimately promoting apoptosis (49, 75–77). Furthermore, during apoptosis, Panx1 releases ATP as “find-me” signals, which attract macrophages to efficiently clear apoptotic cells (75, 78). Studies have indicated that Panx1 participates in Fas-induced apoptosis of leukocytes via the caspase-8/Panx1/P2X_7_R signaling cascade, wherein caspase-8 induces the cleavage of Panx1 and promotes cellular death in Jurkat cells (79). In neurons, elevated levels of Panx1 may exacerbate apoptosis by promoting the TLR2/TLR4/NF-κB pathway (80), mediating Ca^2+^ current, and upregulating the levels of caspase-3 and Bax, a member of the pro-apoptotic BCL-2 (B cell lymphoma-2) protein family, further contributing apoptosis (81). Nevertheless, whether Panx1 expression levels influence the apoptosis level of immune cells requires further investigation.

## 4 Panx1’s regulation of blood flow and implications for microcirculation disorder

Under normal physiological conditions, the body carefully regulates microcirculatory blood flow to ensure adequate oxygen supply to tissues. However, in the context of sepsis, the release of a multitude of pro-inflammatory molecules into the bloodstream disrupts microcirculation, encompassing vessels with a diameter of approximately 100 μm, such as arterioles, capillaries, venules, and micro-lymphatics. This disruption affects various crucial elements involved in microcirculation, including endothelial cells, immune cells, red blood cells, white blood cells, and platelets. Consequently, a significant number of capillaries become dysfunctional, and microcirculatory blood flow undergoes abnormal redistribution, contributing to the establishment of organ dysfunction (82).

**Figure 2:**
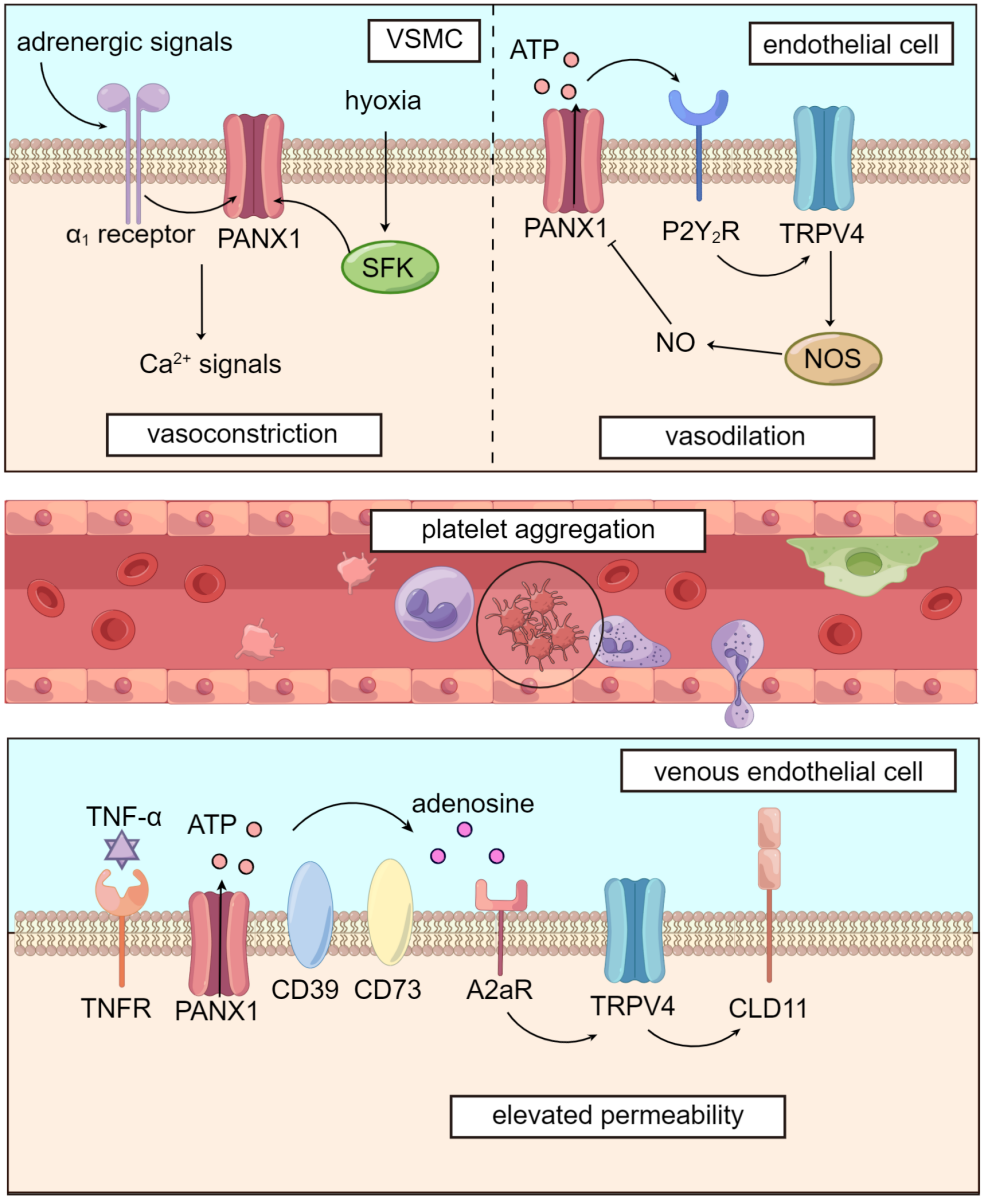
Panx1 mediates blood flow and dampens microcirculation. Panx1 regulates vascular tone in several ways. In VSMCs, adrenergic signals or phosphorylation by SFK opens Panx1 and leads to downstream Ca2+ signals, causing vasoconstriction. In vascular endothelial cells, Panx1 promotes activation of TRPV4 channel via purinergic signaling and enhances activity of NOS, leading to NO-dependent vasodilation. Activation of Panx1 hemichannels, which is widely distributed in the circulatory system, dampens microcirculation by promoting platelet aggregation and elevating venous permeability.

Notably, Panx1is expressed in various cells of the microcirculation, such as vascular smooth muscle cells (83, 84) and vascular endothelial cells (85). The widespread distribution of the Panx1 protein suggests its potential impact on microcirculation in several ways, such as regulation of blood flow, promotion of microcirculatory thrombosis, and modulation of endothelial permeability (**Figure 2**).

### 4.1 Panx1’s role in regulating blood flow

Panx1 plays a significant role in regulating blood flow through its influence on vascular tone. In VSMCs (vascular smooth muscle cells), Panx1 is coupled to α1-adrenergic receptors and located on the caveolae of the cell membrane. Panx1 promotes VSMC contraction, following sympathetic adrenergic signals stimulation, resulting in vasoconstriction and increased blood pressure (83, 84). The inhibition of Panx1 hemichannels using specific inhibitors like mefloquine and probenecid has been shown to reduce blood pressure by attenuating adrenergic-mediated vasoconstriction (83).

Panx1 is also involved in blood flow regulation under hypoxic conditions in the pulmonary circulation. Hypoxia induces the phosphorylation of the Tyr198 site of Panx1 in PASMCs (pulmonary artery smooth muscle cells) by SFK. This activation leads to the opening of Panx1 hemichannels and subsequent Ca^2+^ influx, facilitating pulmonary vasoconstriction and blood transfer from under-ventilated to well-ventilated areas during mechanical ventilation (86). Previous studies have found that TRPV4 (transient receptor potential cation channel subfamily V member 4), a channel that mediates Ca^2+^ influx, is involved in PASMCs’ response to hypoxia and subsequent vasoconstriction (87). However, Panx1 conducts Ca^2+^ influx through a TRPV4-independent pathway (86). Further research is needed to verify the TRPV4-independent mechanism by which Panx1 regulates vascular tone in VSMCs, as interactions between Panx1 and TRPV4 appear to participate in regulating vascular tension, as we will explore in subsequent sections.

Panx1 has been implicated in various signaling pathways in regulating vascular endothelial tension, with a particular focus on the NO (nitric oxide) pathway (88, 89). Studies have shown that Panx1 is closely linked with TRPV4, contributing to lowering pulmonary artery pressure (85). In murine PASMCs, Panx1 hemichannels, P2Y_2_ receptors, and TRPV4 are co-localized on caveolae, forming the Panx1-ATP-P2Y_2_R-PLC (phospholipase C)-PKCα (protein kinase C α)-TRPV4 pathway (84, 90). This signaling cascade leads to NO-mediated pulmonary artery relaxation, as TRPV4 facilitates Ca^2+^ influx, which activates NO synthase in vascular endothelial cells (89). Furthermore, this pathway exhibits a negative feedback mechanism: NO-induced S-nitrosylation of specific sites on the Panx1 protein, namely Cys-40, and Cys-346, inhibits Panx1 opening, downregulating the activity of the signaling pathway (91).

Panx1 may also impact NO production for NO-dependent regulation of vascular tone through inhibitory actions on eNOS (endothelial nitric oxide synthase). By modulating phosphorylation levels at Ser-1177 of eNOS, Panx1 can ultimately lead to reduced NO synthesis (92, 93). Additionally, Panx1 may promote vascular relaxation through NO-independent pathways, such as EDH (endothelium-derived hyperpolarization) regulation, as reported by Gaynullina et al. (94).

Beyond its involvement in vascular smooth muscle cells and endothelial cells, Panx1’s widespread distribution extends to erythrocytes, which also influences vasodilation regulation. Studies suggest that in arterioles, ATP released from erythrocytes reaches higher concentrations in the cell-free layer, activating purinergic signaling pathways in vascular endothelial cells (95). This leads to Ca^2+^ wave propagation and relaxation of upstream VSMCs, ultimately resulting in vessel dilation (10). This supports the notion that hypoxia-induced opening of Panx1 hemichannels dilates blood vessels and modulates tissue perfusion (96). Moreover, erythrocytes may release substantial amounts of ATP in capillaries under the influence of hematocrit. This leads to endothelial cell hyperpolarization, propagating along the capillary network to upstream vessels, thereby regulating oxygen supply (95, 97). This mechanism is reminiscent of the EDH-like regulation described above.

### 4.2 Panx1 in promoting microthrombosis

During sepsis, the immune response triggered by invading pathogens also activates pathways involved in hemostasis. While coagulation activation can promote innate immunity (98), extensive coagulation during sepsis may lead to microcirculation disruption and even disseminated intravascular coagulation (DIC). Panx1, expressed on human platelets and interacting with P2X_1_ receptors, plays a crucial role in platelet aggregation by promoting Ca^2+^ influx (99, 100).

When endothelial cells are damaged, collagen is exposed, activating GPVI (glycoprotein VI) on platelets, which in turn promotes SFK phosphorylation. The interaction between SFK and Panx1 leads to the opening of Panx1 and the subsequent release of ATP. ATP then activates P2X_1_ receptors on platelets, causing a further influx of Ca^2+^ and platelet aggregation (99). Notably, the genetic variation of PANX1 400A>C (rs1138800) encodes a gain-of-function with increased platelet reactivity to collagen in healthy individuals, providing additional evidence for the involvement of Panx1 in inducing platelet aggregation (101).

Furthermore, Panx1 on platelets responds to mechanical stress by releasing ATP, inducing inward Ca^2+^ flow, and promoting platelet aggregation, thereby contributing to arterial thrombosis (99). Deletion of PANX1 prevents the aggregation of platelets (102), underscoring the potential of Panx1 as an inhibitory target for microthrombosis.

Given its role in promoting microthrombosis, Panx1 represents a critical factor in the delicate balance between beneficial coagulation and harmful microcirculation disruption during sepsis. Targeting Panx1 may offer a promising therapeutic approach to mitigate the adverse effects of excessive coagulation and microthrombosis in septic patients. However, further research is needed to fully understand the complex interplay between Panx1, platelet function, and microcirculation in the context of sepsis, ultimately leading to the development of targeted therapies.

### 4.3 Panx1’s impact on endothelial permeability and leakage

During infections, the endothelial barrier is dynamically regulated to allow leukocyte access to tissues through diapedesis, facilitating pathogen clearance. However, sepsis severely compromises the integrity of the endothelial barrier, leading to persistent permeability elevation, leakage, edema, hemodynamic disorder, and potentially respiratory failure. Panx1 has been identified as a key player in increasing vascular permeability, and intriguingly, its effect appears to be contingent on the type of blood vessel involved.

Studies by the same research group revealed that venous endothelial cells express higher levels of Panx1 and are more susceptible to Panx1-induced permeability elevation than arterial endothelial cells (103, 104). In response to stimulation signals like TNF-α (tumor necrosis factor-α), Panx1 hemichannel is activated through SFK-mediated phosphorylation at the Tyr198 site. This activation opens Panx1, leading to the release of ATP. Extracellular ATP is then broken down into adenosine by ectonucleotidases CD39 and CD73. Adenosine binds to A2 receptors, initiating downstream activation of TRPV4 through the cAMP/PKA pathway. Consequently, the arrangement of CLD11 (claudin 11), a major cadherin in endothelial cells, is disrupted, resulting in increased venous permeability. However, it’s important to note that these experiments were conducted on human umbilical vein endothelial cells. A2 receptors may vary across different endothelial cells, such as those in the alveolar microcirculation (105). Therefore, further research is required to clear the role of this Panx1-involved pathway in the microcirculation of specific organs.

In addition to its impact on venous permeability, Panx1 has been implicated in the inflammatory response of microcirculation-associated cells (20), such as pyroptosis, apoptosis, and immune cell chemotaxis, which can contribute to increases vascular permeability. Several potential mechanisms have been proposed for how Panx1 influences vascular permeability: Panx1 can upregulate VCAM1 (vascular cell adhesion molecule 1) on venous endothelium, promoting immune cell chemotaxis and adhesion (104); Panx1-mediated Ca^2+^ influx can trigger the NK-κB cascade, leading to inflammation (106); and Panx1 hemichannels on erythrocytes can be activated by fluid shear, promoting gap formation between endothelial cells, further increasing vascular permeability (107).

While PANX1 is expressed in lymphatic vessels, limited studies suggest a possible association between Panx1 in lymphatic vessels and lipid metabolism and atheromatous plaque development (108). However, there is currently no evidence to indicate that Panx1 is directly involved in the clearance of edematous exudate. The understanding of Panx1’s role in endothelial permeability and leakage is an area of ongoing research, and further investigations are needed to comprehensively unravel its involvement in the complex processes underlying sepsis-induced microcirculation disruption.

## 5 Panx1 in organ dysfunction during sepsis

**Figure 3:**
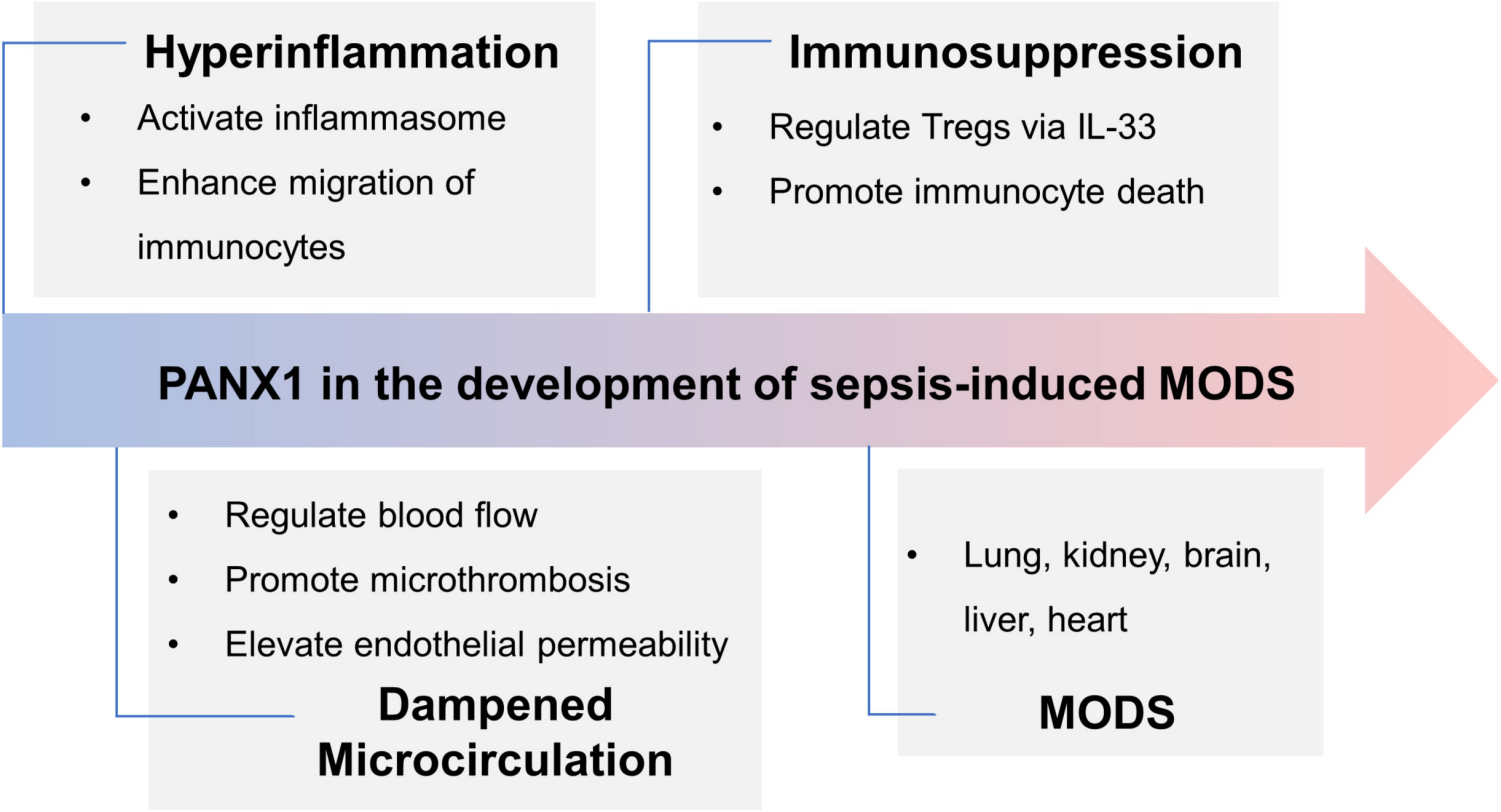
Panx1’s role in the development of sepsis-induced MODS. In the early phase of sepsis, Panx1 contributes to hyperinflammation by activating inflammasome and attracting immunocytes, while it promotes immunosuppression via IL-33-mediated Tregs and immunocyte apoptosis in the late phase. Activation of Panx1 hemichannels dampens microcirculation, aggravating hemodynamic disorder and organ dysfunction. Further, expression of Panx1 in different organs deteriorates injury. Overall, during sepsis, Panx1 contributes to MODS.

Sepsis-3 defines sepsis as a dysregulated host response to infection, often resulting in organ failure (109). The organs commonly affected during sepsis are the lungs, kidneys, liver, heart, and brain. The severity and extent of organ failure can vary from patient to patient, with some experiencing mild dysfunction in one or two organ systems. In contrast, others may face multiple organ failures, posing life-threatening situations. Identifying the specific target organs affected during sepsis is crucial for effective management. In this section, we will focus on Panx1’s involvement in various organs and its role in promoting inflammatory injury recognizing that its functions can differ significantly across different organs (**Figure 3**).

### 5.1 Lungs

Panx1 is expressed on the pulmonary vascular endothelial and epithelial cells (20, 110). Its precise function in lung inflammation remains largely unexplored. Studies have focused on understanding Panx1’s physiological mechanisms in the lungs. For instance, elevated hydrostatic pressure was found to activate Panx1 hemichannels in pulmonary epithelial cells, resulting in the efflux of K^+^ through the purinergic pathway, indicating a potential role for Panx1 in pressure transduction of non-agitated cells (111). Mechanical stress has been implicated in Panx1 hemichannel’s activation, with piezo 1 channel activation leading to the opening of Panx1 hemichannels on alveolar epithelial type I cells and influencing surfactant secretion by alveolar epithelial type II cells (110).

Given this information, it is reasonable to consider Panx1’s potential involvement in the development of ALI (acute lung injury). The integrity of the alveolar-capillary barrier is crucial for normal blood-air exchange, and any damage to this barrier can result in ALI or ARDS (acute respiratory distress syndrome). ALI can be triggered by inhaled pathogens, mechanical stress, and pro-inflammatory molecules (112). Pulmonary circulation plays a vital role, with arterioles, capillaries, and veins collectively contributing to pulmonary vascular resistance. When insulted by factors such as pulmonary endothelial dysfunction, microthrombosis, altered vascular permeability, vasoactive mediator imbalance, hypoxic pulmonary vasoconstriction, and vascular remodeling (113–115), pulmonary vascular pressure may increase, leading to pulmonary vascular dysfunction and contributing to severe diseases like ARDS and sepsis (116, 117).

During inflammation, pro-inflammatory molecules like TNF-α may activate Panx1 hemichannels in the lung, triggering the activation and recruitment of immune cells in the alveolar microcirculation. Simultaneously, mechanical stress arising from improper mechanical ventilation or autonomous respiratory attempts may activate Panx1 hemichannels in the alveolar microcirculation, leading to pyroptosis and further damaging the alveolar-capillary barrier, exacerbating pulmonary edema and promoting an inflammatory response. Although there is limited research on Panx1’s specific role in ALI, a recent study has suggested its potential involvement in pulmonary ischemia-reperfusion injury, pointing toward its potential therapeutic significance in treating ALI (20).

### 5.2 Kidney

Immunodetection studies have provided insights into the distribution of Panx1 within the renal system, revealing its presence in various segments of renal tubules, including proximal tubules, thin descending limbs, and collecting ducts. Additionally, Panx1 expression is observed explicitly in VSMCs of afferent and efferent arterioles within the renal vasculature (118). This diverse expression pattern suggests a potential role for Panx1 in renal functions.

One intriguing aspect of Panx1’s involvement in renal physiology is its implication in ATP-independent control of renin secretion from juxtaglomerular cells, which modulates intracellular Ca^2+^ concentration (119). Moreover, Panx1’s mechanosensitivity has been associated with the development of renal diseases (120).

Under hazardous conditions, such as ischemia-reperfusion injury, Panx1 has been shown to cause damage to renal tubular epithelial cells through the purinergic pathway, leading to the subsequent activation of inflammasome (121). Yin et al. demonstrated that in hypoxia/reoxygenation conditions simulating renal ischemia-reperfusion injury, upregulated caspase-11 cleaves Panx1, triggering inflammasome activation and resulting in cell injury and death. This suggests the potential involvement of the caspase-11/Panx1/NLRP3 pathway in renal I/R (ischemia/reperfusion) injury (21). Panx1’s role in kidney inflammation is also evident in vascular endothelial cells (122). Additionally, Panx1 on macrophages enhances the inflammatory response, with necrotic tubular epithelial cells generating danger signals that activate the inflammasome via the TLR2/caspase-5/Panx1 pathway, leading to the transformation of macrophages into a pro-inflammatory phenotype (44).

Furthermore, it has been demonstrated that Panx1 is involved in ferroptosis following kidney I/R injury (70). Inhibitors of Panx1 hemichannels, such as probenecid, have shown the potential to reduce renal I/R damage, highlighting the significance of Panx1 in the context of AKI (46, 121).

### 5.3 Brain

Panx1 is found in various regions of the CNS, including the cortex, hippocampus, striatum, thalamus, and cerebellum. It is predominantly found in principal excitatory neurons, GABAergic interneurons, and residing immune cells (123). Interestingly, Panx1 expression is not constant throughout life; it is most prominent during early development and gradually diminishes with age, suggesting a role for Panx1 in CNS development (124).

Within the CNS, Panx1 hemichannels play a pro-inflammatory role and can be activated by various factors. For instance, after ischemia, activation of SFK leads to the phosphorylation of Panx1 hemichannel at Tyr-308, resulting in hypoxia-induced depolarization, mitochondrial dysfunction, Ca^2+^ dysregulation, and neuronal death (13, 125). Furthermore, Panx1 hemichannels can be activated by nitrosylation in their intracellular region by NO, leading to neuronal inflammatory damage (91, 126).

In addition to directly causing neuronal injury, Panx1 can influence blood flow regulation and contribute to ischemic injury in the nervous system. Interestingly, vascular endothelial cell-specific PANX1 knockdown, but not VSMCs, has reduced cerebral infarct volume after I/R injury during middle cerebral artery occlusion (127).

During the early phase of inflammation, activation of Panx1 hemichannels can lead to inflammatory cellular death through the purinergic pathway, amplifying the inflammatory response and causing a large-scale release of pro-inflammatory molecules, such as IL-1β and HMGB1 (high mobility group box 1). Panx1 contributes to brain dysfunction in sepsis-induced encephalopathy by promoting pyroptosis (72). The inhibition of Panx1 hemichannels, such as with probenecid, has shown promise in reducing levels of inflammatory factors (e.g., IL-1β, IL-6, and TNF-α) in the CNS, as well as improving memory retention and ameliorating behavioral deficits (128).

These findings collectively highlight the significant role of Panx1 in CNS physiology and pathology, indicating its potential as a therapeutic target for managing neuroinflammatory conditions, specifically in sepsis. Further research is essential to fully comprehend the precise mechanisms and therapeutic implications of Panx1 modulation in the CNS.

### 5.4 Liver

Despite the growing evidence suggesting that Panx1 increases the inflammatory response, its specific role in the liver hasn’t been extensively discussed. However, recent studies have shed light on the significant involvement of Panx1 in liver physiology and pathology.

Panx1 is highly expressed in both murine and human livers, under both healthy and diseased situations (129), and primarily engages in inflammation and immunomodulation within the liver. In non-alcoholic hepatitis, Panx1 has been implicated in mediating the production of IL-1β, contributing to the development of hepatic inflammation (130). Additionally, Panx1 appears to play a role in liver fibrosis through an ATP-dependent mechanism (131, 132).

In models of infection following liver transplantation, Panx1 exhibits immunomodulatory effects through the Panx1-P2X_2_R-IL-33 signaling axis. This leads to increased infiltration of Treg cells, thereby reducing bacterial infection-induced liver injury (66, 67).

Intriguingly, the role of Panx1 in the progression of acute liver failure appears to be sequential. Specifically, PANX1 knockdown effectively reduces liver injury within the first 24 hours, as evidenced by relatively low levels of serum AST (aspartate transaminase) and ALT (alanine aminotransferase). However, beyond the initial 24 hours, PANX1 deletion shows no significant protective effect, and by 48 hours, PANX1 knockdown fails to reduce the necrotic area (133). This suggests that Panx1 may have a time-sensitive impact on inflammatory injury in the liver, and its role in liver pathophysiology is more complex than that of a simple pro-inflammatory regulator.

### 5.5 Heart

Panx1 is widely expressed in the cardiovascular system and is crucial in cardiac rhythm regulation (134). The Cl^-^ permeability of Panx1 determines its contribution to cardiac rhythm. When Ca^2+^ efflux from the sarcoplasmic reticulum activates Panx1 hemichannels on cardiomyocytes, it leads to high conductance. However, in the absence of Ca^2+^ current, episodic opening of Panx1 causes action potentials, potentially resulting in arrhythmias (135).

Panx1 serves distinct roles during acute and chronic heart inflammation, operating through separate signaling pathways. In cases of I/R injury, Panx1 and P2X_7_ receptors are involved in the release of cardioprotectants. Pre- or postconditioning with P2X_7_R agonists, such as ATP, has been shown to reduce the damaged area, indicating that the cardioprotective effects associated with Panx1 and P2X_7_ receptors are likely mediated through the release of ATP. However, the exact mechanism remains unknown (136, 137).

However, in chronic inflammation, such as myocardial fibrosis, ATP via Panx1, combined with P2X_6_ receptors, activates the heterotrimeric G12 family G protein. The activation promotes the expression of fibrogenic genes, leading to myocardial fibrosis (138). The role of Panx1 in the heart during sepsis and its potential therapeutic efficacy remains to be discovered due to a lack of studies in this specific context.

## 6 Pharmacological inhibition of Panx1 hemichannels

There are currently no drugs available that specifically target and inhibit Panx1. Still, some compounds have been reported to exhibit inhibitory effects on Panx1 activity and have been extensively studied in experimental settings. Among these compounds, probenecid, carbenoxolone, and ^10^panx1 have shown promise and potential for future therapeutic approaches (139, 140). Detailed information can be referred to a Review by Koval et al. (141).

Probenecid, a medication commonly used for gout, is clinically used to increase effective concentrations of antibiotics, chemotherapeutics, and other drugs (142). Probenecid has been found to inhibit Panx1 currents in a concentration-response manner (143) and has been widely used to inhibit the transport activity of Panx1 hemichannels. It can suppress inflammasome activity (144), inhibit α-adrenergic receptor-mediated vasoconstriction (145), and enhance microtubule stability (146). Besides Panx1, probenecid broadly inhibits other transport channels and suppresses receptors, such as P2X_7_ receptors, which can limit its application in experiments (47). Nevertheless, probenecid significantly alleviates sepsis-associated damage in experimental settings. Studies have revealed that probenecid can mitigate cerebral I/R injury (147), skeletal muscle cellular energy crisis, and histopathological damage in sepsis (148), as well as promote recovery from renal I/R injury (121). Interestingly, published reports demonstrated that probenecid efficiently inhibits virus replication in cells and murine models (149, 150). Along with its contribution to anti-inflammation, probenecid may be a competent agent in treating SARS-CoV-2-associated sepsis.

Initially developed as an anti-ulcer medication, Carbenoxolone inhibits Panx1 activity by binding to its first extracellular loop of Panx1 (151). It has been demonstrated to inhibit Panx1 hemichannels in various cell types, including neurons, astrocytes, and macrophages (152–154). Although widely used to investigate Panx1’s role in various physiological and pathological processes, its non-specificity and off-target effects may limit its experimental application (155, 156). Carbenoxolone exerts protective impacts in septic conditions (46). The underlying mechanisms may involve its inhibition of Cx hemichannels, which also allows the passage of ATP, and blockage of HMGB1 release (154, 157), in addition to its interference with the Panx1 protein. Carbenoxolone can potentially treat sepsis by alleviating vascular leakage and renal injury in septic conditions (46, 158).

^10^panx1, a 10-amino acid peptide derived from the extracellular loop of the Panx1 protein, provides selective inhibition of Panx1 without affecting ATP-evoked currents, distinguishing it from probenecid and carbenoxolone (159). Studies have shown that ^10^panx1 effectively inhibits Panx1-mediated ATP release and inflammasome activation, reducing inflammatory responses in various experimental models (160–162). However, it’s essential to note that the long-term safety and efficacy of ^10^panx1 as a therapeutic agent for sepsis are still being investigated, as research by Chen et al. has suggested that ^10^panx1 may exacerbate sepsis-induced animal lethality (23).

Though inhibitors of Panx1 show promising outcomes in animal models of sepsis, there is still a long way before clinical application in treating sepsis, as no clinical trials have been reported yet. Moreover, as discussed above, Panx1 involves both hyperinflammation and immunosuppression. As a result, solely targeting it may not be sufficient to address the complexity of sepsis and sepsis-induced MODS. It could worsen immune disorders and organ damage in patients. Therefore, further evidence is required to understand the pattern of Panx1 hemichannel activation in sepsis before exploring its clinical therapeutic potential. More comprehensive research is needed to elucidate Panx1’s role and potential as a therapeutic target in sepsis and sepsis-induced MODS.

## 7 Conclusion

This comprehensive review delves into the potential roles of Panx1 in the pathogenesis of sepsis, examining its contribution to the excessive inflammatory response in the early phase, immunosuppression in the late phase, and impairment of microcirculation. Considering Panx1’s expression in multiple organs and its involvement in organ inflammatory injury, it is reasonable to suggest that Panx1 may play a role in the development of MODS. Inhibiting Panx1 hemichannels emerges as a promising therapeutic strategy against sepsis and sepsis-associated MODS; however, further research is essential before its clinical translation can be realized. As the scientific investigation on Panx1 hemichannels in sepsis is limited, it becomes crucial to explore its distinct functions at different phases of sepsis and understand how it contributes to organ injuries. This makes Panx1 an intriguing potential therapeutic target for sepsis and sepsis-induced MODS, offering a fresh approach to therapeutic interventions against these challenging disorders. Given its significant involvement in various critical processes, Panx1 represents a promising therapeutic target for sepsis and MODS.

## Author contributions

XC wrote the paper, SY proposed the theme of article, LM assisted in writing, and YL and HH provided guidance. All authors contributed to the article and approved the submitted version.
